# Syphilis Not of American Origin

**Published:** 1891-08

**Authors:** 


SYPHILIS NOT OF AMERICAN ORIGIN.—It has been supposed by some syphilograpers that syphilis was brought from America by Columbus' sailors on their return from the discovery of the New World, and that Europe owes this boon, as well as those of tobacco, potatoes and cinchona, to the western hemisphere. Later investigations, however, have pretty well disproved this theory of the origin of the disease, most of the modern authorities attributing the rise of the scourge to a much earlier date. A poem on the "contagious and cursed bubo," written by Francisco Lopes, of Villalobos, in Spain, nearly four hundred years ago, has recently been discovered in the royal library of Madrid, and republished. In this work, published in 1498, and of course written still earlier, the author describes nearly all the lesions now recognized to be those of tertiary syphilis. This would imply that he had seen a great number of cases, and it is hardly probable that there would have been very many cases showing tertiary lesions had the disease been introduded only five years before by Columbus' companions, who returned from their first voyage in 1493.—*Medical Record.*

